# Chemogenetic Activation of RFRP Neurons Reduces LH Pulse Frequency in Female but not Male Mice

**DOI:** 10.1210/jendso/bvae159

**Published:** 2024-09-17

**Authors:** India L Sawyer, Maggie C Evans, Asha Mamgain, Caroline Decourt, Karl J Iremonger, Greg M Anderson

**Affiliations:** Centre for Neuroendocrinology, and Department of Anatomy, University of Otago School of Biomedical Sciences, Dunedin 9016, New Zealand; Centre for Neuroendocrinology, and Department of Anatomy, University of Otago School of Biomedical Sciences, Dunedin 9016, New Zealand; Centre for Neuroendocrinology, and Department of Anatomy, University of Otago School of Biomedical Sciences, Dunedin 9016, New Zealand; Centre for Neuroendocrinology, and Department of Anatomy, University of Otago School of Biomedical Sciences, Dunedin 9016, New Zealand; Centre for Neuroendocrinology, and Department of Anatomy, University of Otago School of Biomedical Sciences, Dunedin 9016, New Zealand; Centre for Neuroendocrinology, and Department of Anatomy, University of Otago School of Biomedical Sciences, Dunedin 9016, New Zealand

**Keywords:** RFRP, RFamide-related peptide, LH pulses, GnRH

## Abstract

**Context:**

The neuropeptide RFRP-3 (RFamide-related peptide-3) is thought to play a role in the negative regulation of fertility. However, the exogenous administration of RFRP-3 yields varying results depending on the dose and route of administration, sex of the subject, and many other variables. Manipulation of in vivo neuronal activity using DREADDs (designer receptor exclusively activated by designer drugs) technology enables investigation of cell type–specific neuronal activation in a manner that better reflects endogenous neuronal activity.

**Objective:**

To test the effects of RFRP neuronal activation on pulsatile luteinizing hormone (LH) secretion.

**Methods:**

We generated mice expressing the stimulatory hM3Dq designer receptor exclusively in RFRP cells using 2 different Cre-loxP–mediated approaches: (1) we bred mice to express hM3Dq in all *Rfrp*-Cre-expressing cells, including some that transiently expressed *Rfrp*-Cre neonatally (RFRP × hM3Dq mice), and (2) we stereotaxically injected Cre-dependent hM3Dq into the dorsomedial nucleus of RFRP-Cre mice to drive hM3Dq expression exclusively in a subpopulation of adult *Rfrp*-Cre neurons (RFRP-AAV-hM3Dq mice). We then investigated the effects of acute hM3Dq activation on LH pulse frequency in RFRP × hM3Dq mice, RFRP-AAV-hM3Dq mice, and their respective controls.

**Results:**

In both female RFRP × hM3Dq and RFRP-AAV-hM3Dq mice, chemogenetic activation of Cre-driven hM3Dq led to a significant 35% to 50% reduction in LH pulse frequency compared with controls, while no differences in pulse amplitude or mean LH concentration were observed. In marked contrast, RFRP activation did not cause any changes to LH pulse dynamics in male mice.

**Conclusions:**

These data show for the first time that activation of neurons that have expressed *Rfrp*, or of a subset of adult RFRP neurons, can independently suppress LH pulsatility in female, but not male mice.

Reproductive function is critical for species survival, yet it is energetically costly and time intensive, particularly in mammals. When environmental, physiological, and/or temporal conditions are not conducive for reproduction, reproductive suppression is advantageous [1]. The neuroendocrine mechanisms involved in suppressing, or deferring, reproductive function are complex and not fully understood. The neuropeptide RFamide-related peptide-3 (RFRP-3) appears to play a key role in mediating adaptive reproductive suppression, particularly in response to stress (eg, [2]).

We have previously shown that female mice whose RFRP cells are chemogenetically inhibited or ablated do not exhibit the stress-induced reduction in luteinizing hormone (LH) pulsatility observed in their wild-type littermates [3]. These data highlight that RFRP cells play a critical role relaying the effects of acute restraint stress to the hypothalamic–pituitary–gonadal (HPG) axis [4]. Furthermore, chronic chemogenetic activation of RFRP neurons caused delayed puberty in male mice and impaired estrous cyclicity in female mice [3]. However, the mechanism(s) whereby RFRP-3 exerts its effects on the HPG axis are not entirely clear, as some limited evidence suggests it acts peripherally on the gonadotropic cells [5] while other evidence suggests it acts centrally by directly targeting the gonadotropin-releasing hormone (GnRH) neurons in the hypothalamus. Furthermore, RFRP-3 in mammals is expressed in the ovaries and testes, where it has been shown to suppress gametogenesis and sex steroid production [6].

The effects of exogenously administered RFRP-3 on LH pulse dynamics are variable [7]. However, exogenous administration of RFRP-3 is unlikely to closely model physiological activation of RFRP neurons, so it is not surprising that different doses, different routes of administration, or differences in gonadal state of the animal yield different RFRP-3-induced effects on LH pulse dynamics. Many of the studies demonstrating RFRP-3-induced reductions in LH pulsatility were performed on ovariectomized females (eg, [8, 9]), which exhibit increased LH levels due to the removal of negative feedback from ovarian steroids. This situation may not accurately represent what endogenous RFRP-3 does in an ovary-intact animal. Furthermore, there is a degree of promiscuity among RFamide peptides and their receptors, with RFRP-3 able to activate the kisspeptin receptor (which excites GnRH neurons), albeit with a Ki value of around 10 000 times that of kisspeptin itself [10]. Indeed, we have shown that the effects of exogenous central RFRP-3 administration are diminished in a kisspeptin receptor knockout mouse [11]. For these reasons, it is important to re-examine the purported suppression of LH pulsatility by RFRP neurons, which is based largely on the fact that intracerebroventricular administration of RFRP-3 reduces LH secretion in some studies [12-14].

With the advent of chemogenetic tools, some of these nonphysiological caveats can now be avoided. We previously demonstrated that ablating or chemogenetically inhibiting RFRP cells prevents the acute restraint stress-induced reduction in LH pulsatility observed in control female mice [3], but the effects of RFRP neuronal activation on LH secretion were not addressed in that study. To further investigate the effects of RFRP neuronal activity on the neuroendocrine reproductive axis, we used 2 different chemogenetic approaches to activate RFRP neurons and investigate their effects on LH pulse dynamics in male and female mice.

## Materials and Methods

### Animal Care and Ethics Approval

Adult female and male mice were group-housed (unless otherwise specified) in individually ventilated cages maintained on a 12-hour light, 12-hour dark cycle with lights on at 06:00 hours. Housing rooms were maintained at 21 ± 1 °C, and mice were given ad libitum access to food and water. All mice were briefly handled daily for at least 2 weeks before blood sampling. Female mice were also assessed daily for estrous cycle stages using vaginal cytology, classified as in [15], to enable the experiments to be conducted on the day of diestrus. All mouse lines used were of predominantly C57BL/6 background strain. All experiments were approved by the University of Otago Animal Ethics Committee.

### Generation and Validation of RFRP × hM3Dq and RFRP-AAV-hM3Dq Knock-in Mice

To generate RFRP × hM3Dq mice, the F1 progeny of the Cre-dependent hM3Dq mouse line B6N;129-Tg(CAG-CHRM3*,-mCitrine)1Ute/J (heterozygous, obtained from Jackson Laboratories; RRID:IMSR_JAX:026220 https://www.jax.org/strain/026220) [16] and *Rfrp*-Cre mice (B6(Cg)-*Npvf^tm1.1(icre)Gand^*) (heterozygous, MGI:7439911 https://www.informatics.jax.org/strain/MGI:7439911) [3] were used. RFRP × hM3Dq female and male mice (heterozygous for both mutations) and their *Rfrp*-Cre siblings not inheriting the hM3Dq gene (referred to as control mice) were generated and used between 12 and 15 weeks of age. The *Rfrp*-Cre line was genotyped using generic Cre primers (forward: 5′-CCT GGA AAA TGC TTC TGT CCG-3′; reverse: 5′-CAG GGT GTT ATA AGC AAT CCC-3′; annealing temperature 55 °C; product size indicating the Cre allele: 392 bp). The Cre-dependent hM3Dq mice were identified using the following polymerase chain reaction primer sets and annealing temperatures: 5′-AAG GGA GCT GCA GTG GAG TA-3′ (wild-type forward primer), 5′-CCG AAA ATC TGT GGG AAG TC-3′ (wild-type reverse primer), 5′-ATG TCT GGA TCC CCA TCA AG-3′ (mutant forward primer), 5′-GAT GTT GCC GAT GAT GGT CAC-3′ (mutant reverse primer); annealing temperature 55 °C; product size indicating the floxed and wild-type alleles: 440 and 300 bp, respectively. The coexpression of the hM3Dq receptor within RFRP neurons in RFRP × hM3Dq mice and activation of these neurons following clozapine N-oxide (CNO) has been reported previously by our group [3].

For the adeno-associated viruses (AAV)-driven approach to express hM3Dq (referred to as RFRP-AAV-hM3Dq mice), *Rfrp*-Cre female and male mice and their control littermates (not expressing Cre) were bilaterally injected at 9-12 weeks of age with 500 nL of AAV2-hSyn-DIO-hM3D(Gq)-mCherry containing viral particles (2.6 × 10^13^ genome copies/mL in PBS) (Addgene plasmid #44361; RRID:Addgene_44361 https://www.addgene.org/44361/) [17] into the dorsomedial nucleus of the hypothalamus (DMH) (0.4 mm medial and 1 mm posterior to bregma– and 5.4 mm ventral to the skull surface), coordinates guided by The Mouse Brain in Stereotaxic Coordinates [18]. Stereotaxically injected mice were left for 3 weeks following AAV injection, to recover and for viral transfection to occur.

To verify colocalization of RFRP neurons with the DREADD and neuronal activation following treatment with CNO in RFRP × hM3Dq mice, they were given CNO at 1 mg/kg subcutaneously (SC) or vehicle, and after 1 hour were overdosed with 250 mg/kg of sodium pentobarbital, and perfused through the heart with 4% paraformaldehyde (PFA) in 0.1 M phosphate-buffered saline (PBS) pH 7.4. The brains were collected and postfixed in PFA before being transferred to 30% sucrose solution. Coronal sections from the DMH were cut at 30 μm in series of 4. Dual labelled fluorescence or chromogen immunohistochemistry was performed on free-floating sections. For dual immunofluorescence to colocalize the hM3Dq reporter mCitrine and RFRP-3, antibodies were applied in cocktail for mCitrine (chicken anti-GFP 1:5000, Aves lab RRID:AB_2307313 https://scicrunch.org/resolver/AB_2307313, followed by Alexa Fluor 488 goat antichicken RRID:AB_2534096 https://scicrunch.org/resolver/AB_2534096) and for RFRP-3 (rabbit antisparrow GnIH antibody Pac123a 1:5000, kindly provided by Dr George Bentley, University of California Berkeley RRID:AB_2531898 https://scicrunch.org/resolver/AB_2531898, followed by AlexaFluor-568 goat antirabbit RRID:AB_143157 https://scicrunch.org/resolver/AB_143157). To confirm neuronal activation in hM3Dq-DREADD expressing cells, dual chromogen staining for the hM3Dq reporter hemagglutinin (HA) (rabbit anti-HA-Tag C29F4 1:500, Cell Signaling, RRID:AB_1549585 https://scicrunch.org/resolver/AB_1549585 followed by biotinylated goat antirabbit 1:1000, Vector Laboratories BA-1000 RRID:AB_2313606 https://scicrunch.org/resolver/AB_2313606, Vector Elite avidin–biotin complex solution, Vector Laboratories, and 0.5% DAB and hydrogen peroxide solution until brown staining was observed) and the neural activity marker cFos (rabbit anti cFos 1:5000, Santa Cruz Biotechnology, sc-52; RRID:AB_2106783 https://scicrunch.org/resolver/AB_2106783, followed by HRP-conjugated goat antirabbit IgG 1:500, Dako, RRID:AB_2617138 https://scicrunch.org/resolver/AB_2617138, and nickel-enhanced DAB and hydrogen peroxide solution until a blue-black nuclear staining was observed).

To verify colocalization of RFRP neurons with hM3Dq and neuronal activation in RFRP-AAV-hM3Dq mice, *Rfrp*-Cre mice were bred with Cre-dependent tau-green fluorescent protein (τGFP) reporter mice (Gt(ROSA)26Sortm1(CAG-Mapt/GFP)Uboe, MGI ID:4878874 https://www.informatics.jax.org/allele/MGI:4878874) [3] to generate mice exhibiting τGFP exclusively in Cre-expressing cells. The mice were given CNO at 2 mg/kg SC or vehicle, and after 1 hour were overdosed with sodium pentobarbital and perfused for serial brain section collection as described above. Dual labelled fluorescence immunohistochemistry was performed on free-floating sections, in series for the AAV reporter mCherry (chicken anti-mCherry 1:10000, Abcam RRID:AB_2722769 https://scicrunch.org/resolver/RRID:AB_2722769, followed by Alexa Fluor 568 goat anti-chicken RRID:AB_2534098 https://scicrunch.org/resolver/RRID:AB_2534098) and the *Rfrp*-Cre reporter τGFP (chicken anti-GFP 1:5000, Aves lab RRID:AB_2307313 https://scicrunch.org/resolver/AB_2307313, followed by Alexa Fluor 488 goat anti-chicken RRID:AB_2534096 https://scicrunch.org/resolver/AB_2534096) or in cocktail for mCherry and the neural activity marker cFos (rabbit anti-cFos 1:5000, Abcam RRID:AB_2737414 https://scicrunch.org/resolver/AB_2737414), followed by Alexa Fluor 568 goat antichicken RRID:AB_2534098 https://scicrunch.org/resolver/RRID:AB_2534098 and Alexa Fluor 488 goat antirabbit RRID:AB_2630356 https://scicrunch.org/resolver/AB_2630356. All primary antibodies were validated by absence of staining outside the DMH or in tissues not expected to express the target proteins. Stained sections were mounted onto gelatin-coated slides and cover-slipped using Fluoromount (Invitrogen) or DPX mounting medium.

The injection sites of all AAV-injected mice were visually assessed and mapped to their location within the brain. Injections falling within the range of −1.5 and −2.3 relative to bregma were included as RFRP neurons are found throughout this range [3]. Both unilateral and bilateral hits were included. Only mice with transfection in and around the DMH according to these criteria were included in the dataset (1 RFRP-AAV-hM3Dq mouse was excluded based on these criteria).

### Handling Habituation, CNO Administration, and Serial Tail Blood Sampling

From 16-20 weeks of age, the mice were handled daily for at least 2 weeks prior to blood sampling experiments. This handling procedure was designed to habituate the mice to the serial tail-tip bleeding procedure. The mice tails were handled and stroked to simulate blood sampling, and injections were simulated by poking the skin behind the hind legs.

To determine whether pulsatile secretion of LH is affected by stimulation of RFRP neurons, diestrus female and male mice were injected with CNO (2 mg/kg SC) at 09:00 hours. Just after the injection, 1 mm was cut from the end of the tails. Thirty minutes later repeated tail-tip blood samples were obtained from mice every 6 minutes between 09:30 and 12:30 hours. The tails were stroked to stimulate bleeding, and 5 μL of blood per sample was obtained using a pipette tip. Blood was immediately added to 50 μL of PBS-Tw buffer, then rapidly frozen on dry ice. Three control mice and 1 RFRP-AAV-hM3Dq mouse were resampled 2 weeks later because they did not pulse on their first bleed, and 3 RFRP-AAV-hM3Dq mice were resampled due to a failed enzyme-linked immunosorbent assay (ELISA) plate read.

**Figure 1.**
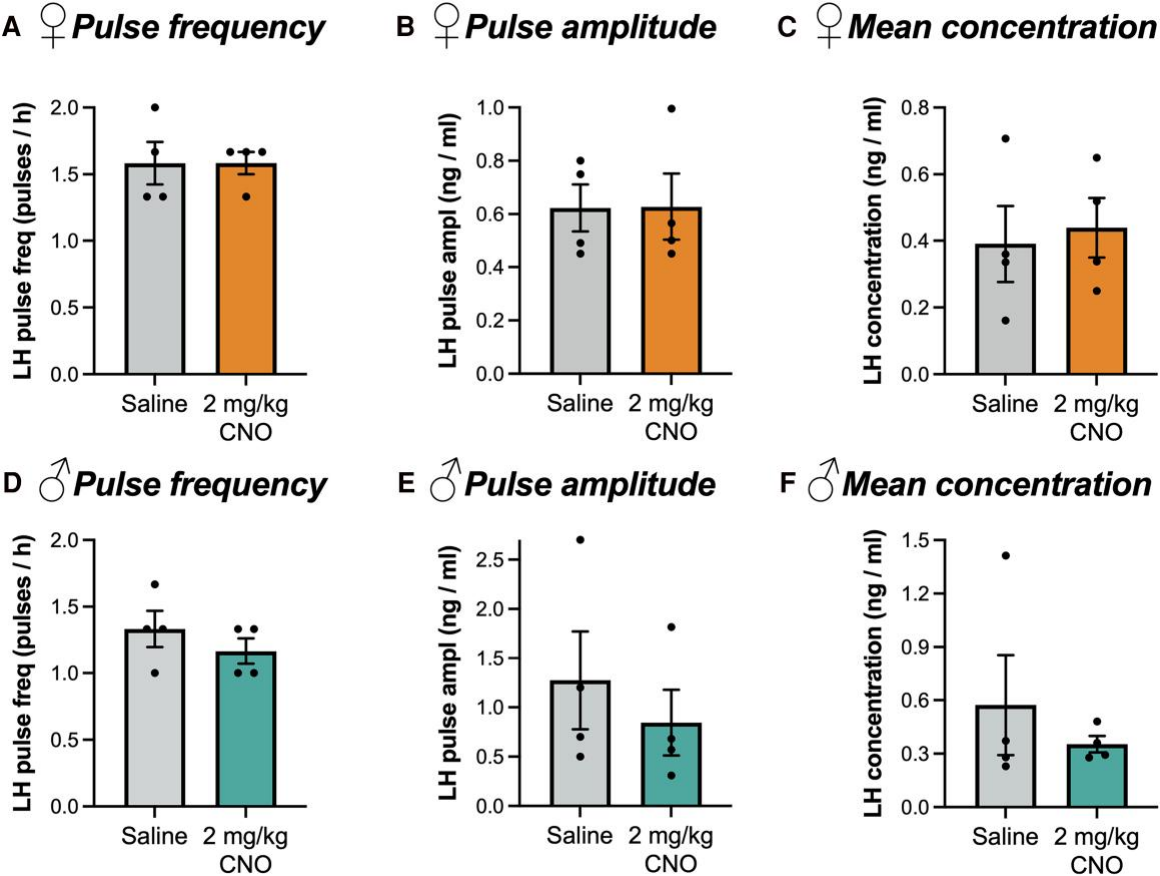
Treatment of wild-type male and female mice with 2 mg/kg CNO does not affect pulsatile LH secretion. Pulse frequency (A, D), average concentration (B, E) and pulse amplitude (C, F) of female mice (A-C) and male mice (D-F) (n = 4). Pulse frequency data analyzed using a Mann–Whitney test; pulse amplitude and LH concentration data analyzed using Student's t-test.

Treatment of ovariectomized female mice which do not express DREADDs with 1 mg/kg CNO has previously been shown to have no effect on pulsatile LH release [19]. To confirm this lack of nonspecific effects in gonad-intact male and female mice at the maximal dose used in this study, a pilot study was conducted in which wild-type C57BL/6 mice were treated with either CNO (2 mg/kg) or saline vehicle. Thirty minutes later they were sampled for LH every 6 minutes for 3 hours as described above. No effect of CNO was observed on LH pulse frequency, average pulse amplitude, or average concentration in either sex (*P* > .4; Mann–Whitney or Student t-tests; Fig. 1). The results of this this pilot study confirmed that 2 mg/kg CNO does affect LH secretion in the absence of DREADDs. Nevertheless, in all LH pulse experiments involving activation of RFRP neurons, mice not expressing hM3Dq but treated with CNO were used as controls.

### LH Pulse Analysis

Diluted whole-blood LH concentrations were assessed in unicate 50-µL aliquots using a sensitive in-house sandwich ELISA, as previously described [20] and recently modified [21]. The assay sensitivity averaged 0.1 ng/mL after correction for the 30-fold sample dilution, and the interassay and intra-assay coefficients of variation were <10%. Control and RFRP-activated data from each experiment were generated at the same time and balanced appropriately across LH ELISA plates. The RFRP × hM3Dq and RFRP-AAV-hM3Dq experiments, as well as the male and female data within the experiments, were generated in different runs. LH pulses were determined using the following criteria: a pulse must increase by 150% within 2 samples, it must have at least 2 data points higher than the preceding and following nadir (except where a clear peak was evident at the first sample, which were included to avoid pulse frequency underestimation), and the pulse amplitude must be higher than 0.2 ng/mL (ie, 2 times the assay sensitivity) [3, 22, 23]. Occasionally, pulse amplitude values (peak concentration minus preceding nadir concentration) were not able to be generated, either because no pulses were detected during the sampling period or because the only pulse detected had a peak at time 0 (so the preceding nadir was unknown). The RFRP-AAV-hM3Dq were also analyzed using the PULSAR algorithm [24], yielding almost exactly the same results (data not shown).

### Statistical Analyses

Statistical analyses were performed and data graphed using Prism software 9.0 (GraphPad). All data are graphed and presented in text as mean ± SEM. For some experiments, descriptive statistics only were performed on the data. We did not consider sex to be an experimental treatment in itself, hence our analyses in males and females were done separately. Pulse frequency data were analyzed using a Mann–Whitney test. For all other data, a Student t-test was used to compare 2 groups and a 1-way analysis of variance with Holm–Šídák's multiple comparisons post hoc analysis was used to compare 3 groups. *P* < .05 was considered to be statistically significant.

## Results

### Validation of Mice

**Figure 2.**
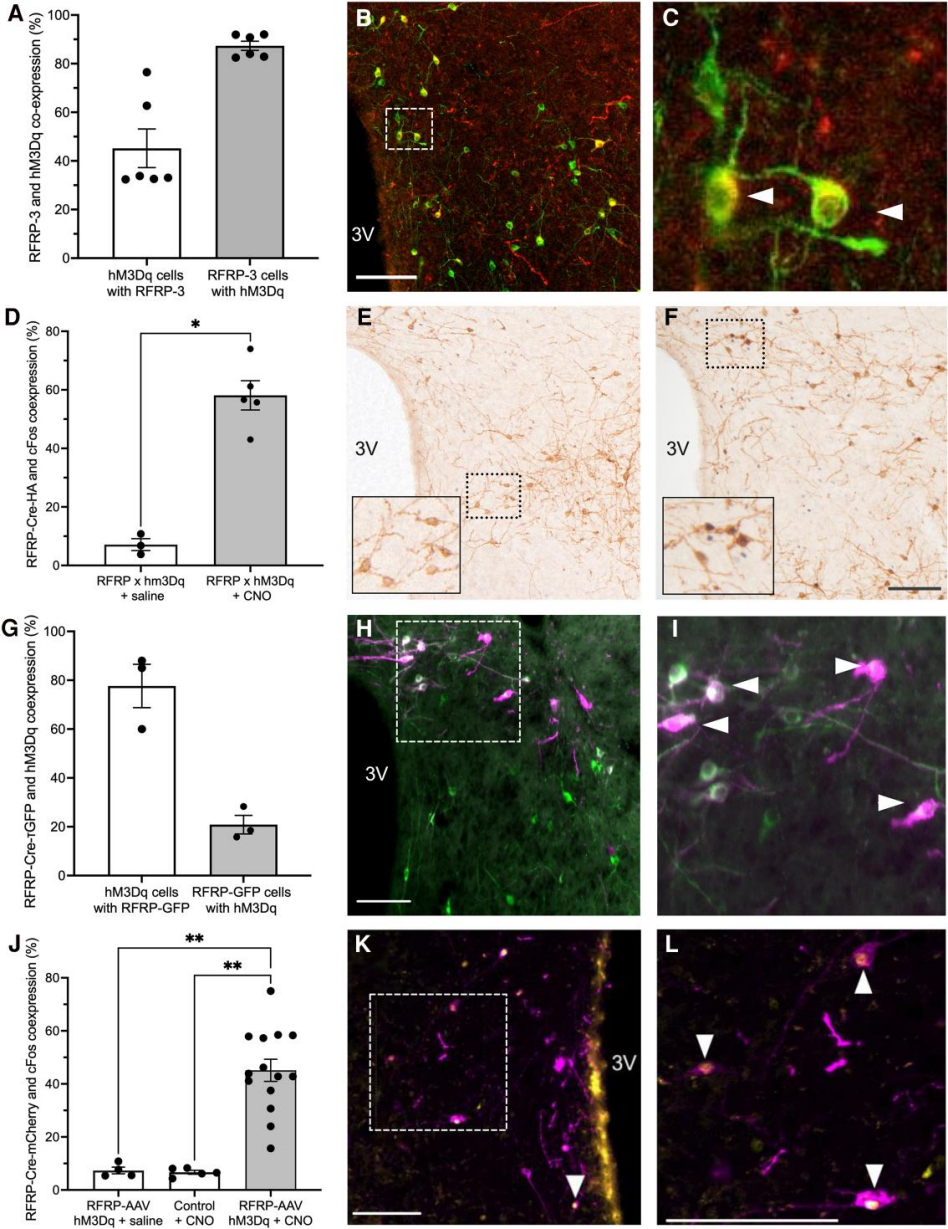
Validation of DREADD expression in *RFRP × hM3Dq* and *Rfrp*-Cre:τGFP-AAV mice. (A) RFRP-3 neurons co-express hM3Dq in RFRP *×* hM3Dq mice, n = 6, 4F, 2M. The percentage of hM3Dq (citrine reporter)-expressing neurons that are also immunoreactive for RFRP-3 and the percentage of RFRP-3 immunoreactive neurons that coexpress hM3Dq are ashown. (B) Representative example image of dual immunofluorescence for RFRP-3 (labelled red) and the hM3Dq reporter mCitrine (green GFP label), with coexpressed cells appearing yellow. (C) Enlargement of the dashed box region in panel B, with co-labelled cells indicated by arrowheads. (D) hM3Dq-DREADD neurons are activated (as indicated by cFos coexpression) following CNO treatment (1 mg/kg SC) in RFRP *×* hM3Dq mice. RFRP × hM3Dq + CNO, n = 5; RFRP *×* hM3Dq + saline, n = 3. Data analyzed using a Mann–Whitney U test. Immunohistochemistry showing hM3Dq as reported by HA staining (brown cytoplasmic stain), and cFos (black nuclear stain) in saline (E) or CNO (F) treated mice. Enlarged insets of dashed box regions show cFos negative (E) or cFos colabeled (F) neurons. (G) RFRP neurons coexpress hM3Dq in RFRP-AAV-hM3Dq mice, n = 3, 1F, 2M. The percentage of hM3Dq (mCherry reporter)-expressing neurons that coexpress the RFRP reporter τGFP and the percentage of RFRP-τGFP neurons that coexpress hM3Dq are shown. (H) Representative example image of dual immunofluorescence for RFRP-τGFP (displayed in green) and the hM3Dq reporter mCherry (displayed in magenta), with coexpressed cells appearing white. (I) Enlargement of the dashed box region in H, with colabeled cells indicated by arrowheads. (J) hM3Dq-DREADD neurons are activated (as measured by cFos co-expression) following CNO treatment (2 mg/kg SC) in RFRP-AAV-hM3Dq mice. RFRP-AAV-hM3Dq + CNO, n = 14; RFRP-AAV-hM3Dq + saline, n = 4; control + CNO, n = 5. Data analyzed using Kruskal–Wallis with Dunn's multiple comparison testing. (K) Representative example image of dual immunofluorescence for hM3Dq-mCherry (magenta) and cFos (displayed in yellow), with coexpressed cells appearing white. (L) Enlargement of the dashed box region in K, with colabeled cells indicated by arrowheads. Data are mean ± SEM. Scale bars = 100 μm. *P < .05; **P < .01. The data in D have been reported previously [3].

The RFRP × hM3Dq mice used in this study were previously validated in [3]. In the current study, 87 ± 2% of RFRP-3 expressing neurons colocalized the hM3Dq-DREADD, while 45 ± 8% of hM3Dq expressing neurons colocalized RFRP-3, reflecting early-life Cre-mediated hM3Dq expression in neurons that no longer produce RFRP-3 during adulthood (n = 6, Fig. 2A; representative immunohistochemistry example shown in Fig. 2B and 2C). RFRP × hM3Dq mice treated with CNO 1 hour prior to sacrifice showed significantly greater hM3Dq neuronal activation as measured by cFos than RFRP × hM3Dq mice treated with saline (58.1 ± 5.0%, n = 5 vs 7.1 ± 2.0%, n = 3, Fig. 2D; representative immunohistochemistry example shown in Fig. 2E and 2F). To verify colocalization of RFRP neurons with AAV-hM3Dq-mCherry, a separate cohort of mice had to be used to enable fluorescent RFRP neuron visualization. By injecting AAV-hM3Dq into the DMH of *Rrfp*-Cre mice also expressing a Cre-dependent τGFP reporter, the hM3Dq receptor was limited much more specifically to RFRP neurons than was the case in RFRP × hM3Dq mice, with 77.7 ± 8.9% of hM3Dq expressing neurons colocalizing *Rfrp*-Cre-τGFP (n = 3, Fig. 2G; representative immunohistochemistry example shown in Fig. 2H and 2I). However, only 20.9 ± 3.8% of *Rfrp*-Cre expressing neurons colocalized the hM3Dq receptor (Fig. 2G), indicating that while hM3Dq-DREADD expression was specific to RFRP neurons, it was only expressed in a subpopulation of RFRP neurons. RFRP-AAV-hM3Dq mice treated with CNO 1 hour prior to sacrifice showed significantly greater hM3Dq neuronal activation as indicated by cFos than RFRP-AAV-hM3Dq mice treated with saline (45.1 ± 4.2%, n = 14 vs 7.4 ± 1.3%, n = 4, Fig. 2J) or Cre negative AAV-injected mice treated with CNO (6.7 ± 0.7%, n = 5, Fig. 2K). In Cre negative AAV-injected mice, mCherry staining was minimal and faint, and limited to the cell bodies. In contrast, the strongly expressed mCherry seen in *Rfrp*-Cre positive AAV mice was in both cell bodies and projections (Fig. 2H and 2I). Visual assessment of the injection site confirmed the RFRP × hM3Dq-AAV mice were injected within the target range of −1.5 and −2.3 relative to bregma [3] in all but 1 case (which was excluded). For the 6 stimulated female and 8 stimulated male RFRP × hM3Dq-AAV mice included in the study, there was an even distribution of rostral DMH (between −1.5 and −1.7 mm relative to bregma), mid-DMH (−1.75 to −2.05 mm from bregma) and caudal DMH (−2.1 to −2.3 relative to bregma) injections sites, with no obvious difference in results obtained between these regions.

### Effect of CNO on LH Pulsatility in RFRP × hM3Dq Mice

**Figure 3.**
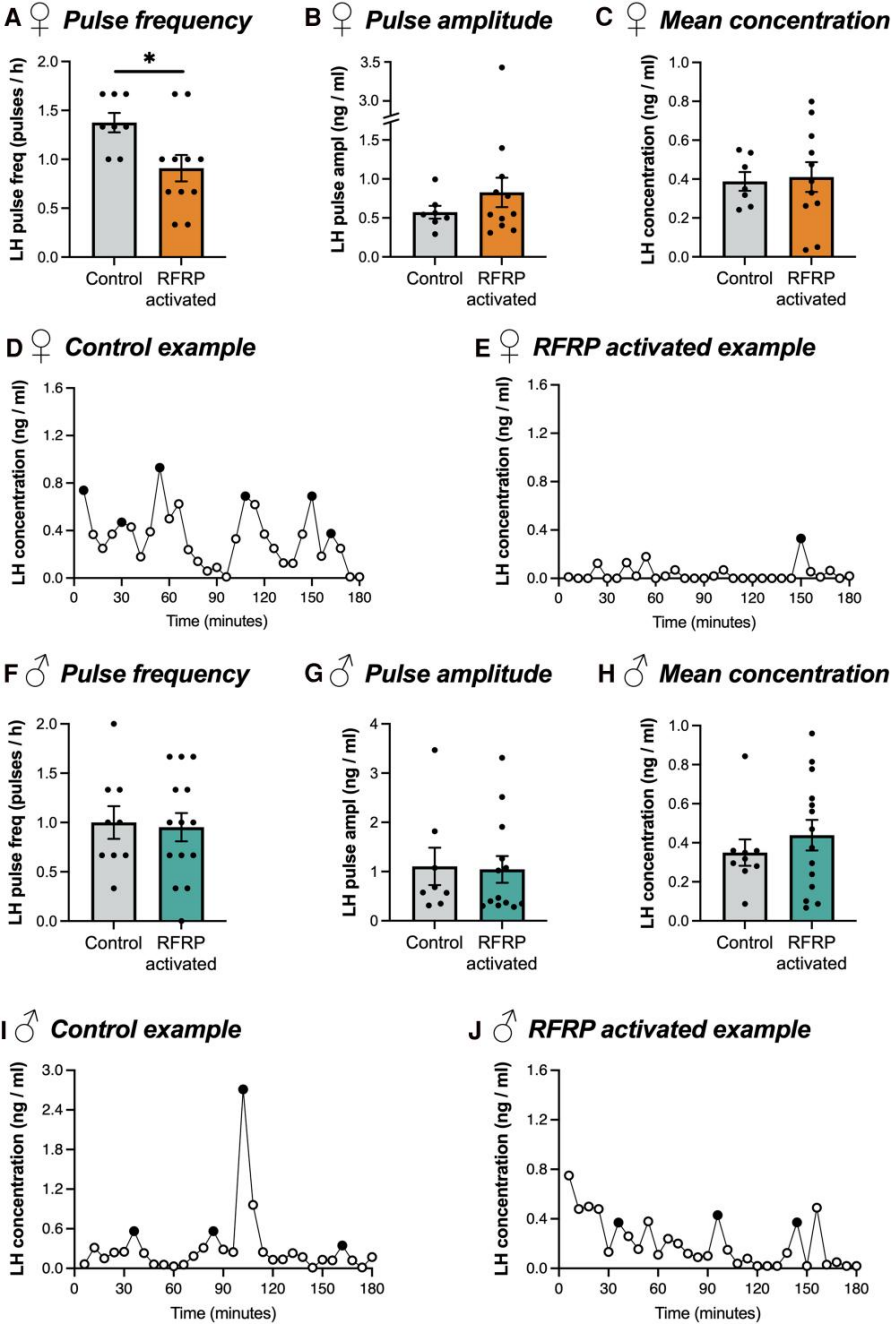
Activation of neurons that have expressed *Rfrp* reduces LH pulse frequency in female but not male RFRP *×* hM3Dq mice. LH pulse frequency (A, F), pulse amplitude (B, G), and average concentration (C, F) of female (A-E, n = 8 control, 11 RFRP *×* hM3Dq) and male (F-J, D-F, n = 9 control, 14 RFRP *×* hM3Dq) mice. Pulse amplitude data were not obtained from 1 female control, 1 male control, and 1 male RFRP-activated mice. All animals were treated 30 minutes prior to sampling with 2 mg/kg CNO. Pulse frequency data analyzed using a Mann–Whitney test; pulse amplitude and LH concentration data analyzed using Student t-tests. Representative examples of LH pulse profiles from control (D, I) and RFRPs-AAV-hM3Dq (E, J) mice. Filled circles indicate the peaks of identified pulses. *P < .05.

To determine the effect of chemogenetically activating all RFRP-Cre–expressing cells on LH pulsatility, male and female control and RFRP × hM3Dq mice were treated with 2 mg/kg CNO subcutaneously and serial blood sampling was performed over a 3-hour period. Consistent with our hypothesis that RFRP neuronal activation acts as a brake on the neuroendocrine reproductive axis, female RFRP × hM3Dq mice exhibited a significant reduction in LH pulse frequency over the 3-hour sampling period compared with control females (Fig. 3A, 0.9 ± 0.13 vs 1.4 ± 0.10 pulses/hour); however no differences in average pulse amplitude (Fig. 3B, 0.92 ± 0.27 vs 0.57 ± 0.08 ng/mL, *P* = .336) or average LH concentration (Fig. 3C, 0.41 ± 0.08 vs 0.39 ± 0.05 ng/mL, *P* = .833) were observed. In contrast to females, no differences in LH pulse frequency were observed between CNO-treated RFRP × hM3Dq and control male mice (Fig. 3F, 0.9 ± 0.13 vs 1.0 ± 0.13 pulses/hour, *P* = .870). Similarly, no differences in or average pulse amplitude (Fig. 3G, 1.04 ± 0.27 vs 1.11 ± 0.38 ng/mL, *P* = .893) or average LH concentration (Fig. 3H, 0.44 ± 0.08 vs 0.35 ± 0.08 ng/mL, *P* = .457) were observed between RFRP × hM3Dq and control male mice. The LH pulse profiles of representative CNO-treated RFRP × hM3Dq males and females can be seen in Fig. 3D, 3E, 3I, and 3J.

### Effect of CNO on LH Pulsatility in RFRP-AAV-hM3Dq Mice

**Figure 4.**
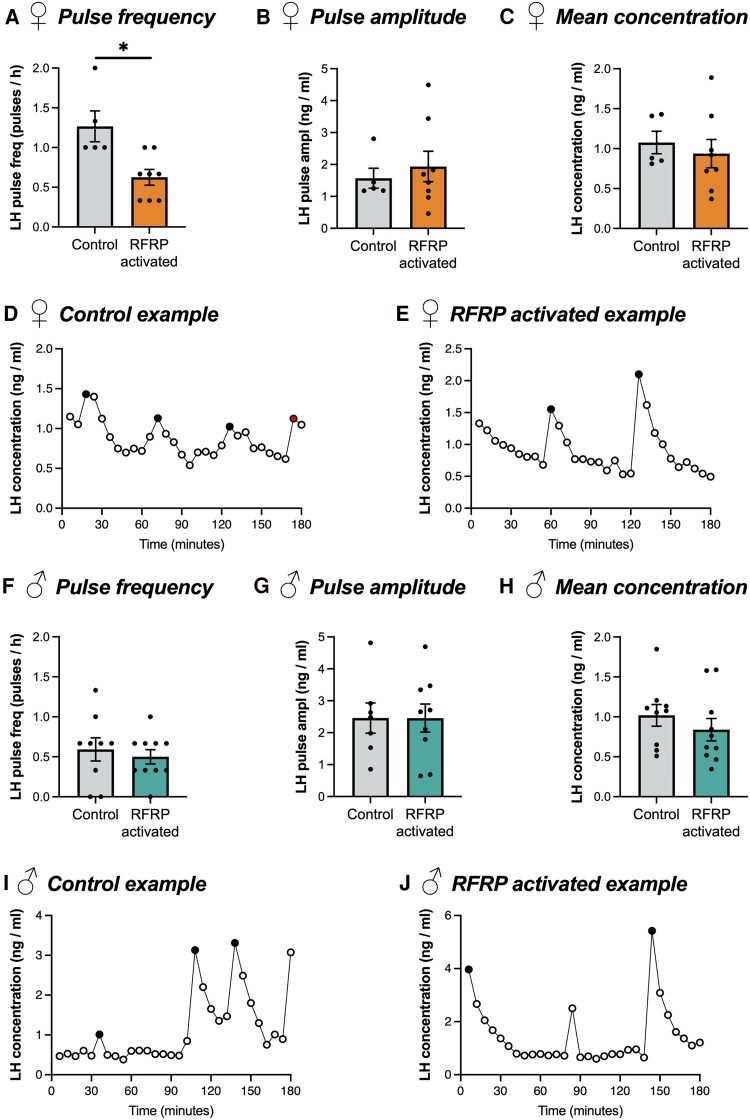
Activation of a subset of adult RFRP neurons reduces LH pulse frequency in female but not male RFRP-AAV-hM3Dq mice. LH pulse frequency (A, F), pulse amplitude (B, G), and average concentration (C, F) of female (A-E, n = 5 control, 8 RFRP-AAV-hM3Dq) and male (F-J, n = 9 control, 10 RFRP-AAV-hM3Dq) mice. Pulse amplitude data were not obtained from 2 male control and 1 male RFRP activated mice. All animals were treated 30 minutes prior to sampling with 2 mg/kg CNO. Pulse frequency data analyzed using a Mann–Whitney test; pulse amplitude and LH concentration data analyzed using Student t-tests. Representative examples of LH pulse profiles from control (D, I) and RFRPs-AAV-hM3Dq (E, J) mice. Filled circles indicate the peaks of identified pulses. *P < .05.

To determine the effect of chemogenetically activating AAV-transfected RFRP-Cre-expressing neurons on LH pulsatility, male and female control and RFRP-AAV-hM3Dq mice were treated with 2 mg/kg CNO subcutaneously and serial blood sampling was performed over a 3-hour period. Consistent with our findings from RFRP × hM3Dq vs control mice, RFRP-AAV-hM3Dq female mice exhibited a significant reduction in LH pulses over the 3-hour sampling period compared to control females (Fig. 4A, 0.6 ± 0.20 vs 1.3 ± 0.10 pulses/3 hours), while also exhibiting no differences in average pulse amplitude (Fig. 4B, 1.94 ± 0.48 vs 1.57 ± 0.31 ng/mL, *P* = .590) or average LH concentration (Fig. 4C, 0.94 ± 0.18 vs 1.08 ± 0.14 ng/mL, *P* = .595). Similarly, no differences in LH pulse frequency (Fig. 4D, 0.5 ± 0.09 vs 0.6 ± 0.14 pulses/3 hours, *P* = .629), average pulse amplitude (Fig. 4G, 2.46 ± 0.44 vs 2.46 ± 0.47 ng/mL, *P* = .998), or average LH concentration (Fig. 4H, 0.84 ± 0.14 vs 1.02 ± 0.14 ng/mL, *P* = .371) were observed between RFRP-AAV-hM3Dq and control male mice, which is consistent with our findings from RFRP × hM3Dq vs control mice. The LH pulse profiles of representative CNO-treated RFRP-AAV-hM3Dq males and females can be seen in Fig. 4D, 3E, 3I, and 3J.

## Discussion

Although RFRP neurons have been considered to be negative regulators of GnRH function since their discovery in 2000 [25, 26], obtaining consistent data regarding their effects on LH pulses has proved elusive [7]. Mildly suppressive effects of centrally-administered neuropeptides under negative feedback conditions when LH levels are already low are challenging to resolve. Even in gonadectomized animals, where LH pulses are unrestrained, suppressive effects of RFRP-3 are not always demonstrable [11-13, 22]. The same is true for glucocorticoid-induced LH pulse suppression, which is only observed in female mice under conditions of estrogenic negative feedback [27]. In the present study we chemogenetically activated RFRP cells in ovary-intact mice (which, despite their negative feedback environment, must still retain capacity for further LH suppression in response to inhibitory stimuli to allow for adaptive reproduction suppression [2]) to investigate the effects of in vivo activation of RFRP neurons on the neuroendocrine reproductive axis, and we demonstrate for the first time that endogenous RFRP activation reduces LH pulsatility in female, but not male, mice. To do this, we used Cre-lox transgenics to express the stimulatory hM3Dq-DREADDs into *Rfrp*-Cre cells, using 2 different approaches, and then used the receptor ligand CNO to endogenously activate them. The first approach we used, crossing *Rfrp*-Cre and Cre-dependent hM3Dq mice, transduces essentially all Cre-expressing cells (87% of RFRP-3 expressing neurons colocalized the hM3Dq-DREADD in the present study). This represents a population of cells that expressed the *Rfrp* gene at 1 point during development. Due to the reduction in *Rfrp* expression from birth to adulthood [28], this population of targeted cells includes some that no longer express *Rfrp*, but Cre-dependent genes continue to be expressed (hence only 45% of hM3Dq expressing neurons colocalized RFRP-3). The second approach we used only transfected Cre-positive neurons still expressing *Rfrp* in adulthood. While this approach more specifically targeted adult RFRP neurons (78% of hM3Dq expressing neurons colocalized *Rfrp*-Cre), it only transfected 21% of the adult RFRP population due to the limited special spread of the injected virus. These approaches therefore have reciprocal limitations. One overexpresses hM3Dq relatively nonspecifically and the other underexpresses it specifically—yet they both resulted in remarkably consistent effects, with activation of the transfected cells causing a reduction in LH pulse frequency in female, but not male, mice. Due to the consistency in findings from the 2 approaches, we have concluded that in vivo activation of RFRP neurons can reduce LH pulse frequency in ovary-intact female mice.

While RFRP-3 is already recognized as a negative regulator of reproductive function (eg, [12, 29, 30]), the mechanism(s) whereby it targets the HPG axis have remained relatively unclear. It has previously been shown that exogenous RFRP-3 can modulate HPG axis activity, but data has been inconsistent as to whether RFRP-3 is acting centrally or peripherally [5, 7, 12]. The fact that RFRP neuronal activation suppressed LH pulse frequency but not pulse amplitude in the current study supports a central mode of action on the LH pulse generator, rather than a pituitary suppression of LH release in response to GnRH. It also remains uncertain whether or not RFRP-3 is exclusively inhibitory to GnRH/LH, as some data suggests it can be stimulatory in in male rodents [11, 31, 32]. In the current study, no evidence of LH stimulation in response to RFRP neuronal activation was observed. One possible explanation is the administration of exogenous RFRP-3 is not physiological. RFRP-3 can weakly bind to the kisspeptin receptor, such that supraphysiological doses of RFRP-3 can stimulate LH release in part due to activation of the kisspeptin receptor [11]. To our knowledge, this is the first study investigating the effects of activation of RFRP neurons on GnRH/LH pulsatility in gonad-intact male and female mice. CNO-hM3Dq-induced RFRP neuronal activation presumably induces release of RFRP-3, but other neuropeptides of neurotransmitters that might be coreleased are unknown at the present time, other than that these neurons have minimal if any colocalization with GABA [33].

These findings build on previous data showing chemogenetic inhibition or ablation of RFRP-Cre-expressing cells can prevent the reduction in LH pulse frequency due to acute restraint stress in females, but not males [3]. While no effect of either chronic RFRP neuronal ablation or acute RFRP neuronal silencing was observed on LH pulse frequency in unstressed mice in that study (implying that other circuits such as estrogen-regulated arcuate kisspeptin neurons predominate to control GnRH and LH under normal conditions), RFRP-ablated or silenced mice did not display stress-induced pulse suppression. This highlights that RFRP neurons play a critical role in mediating the stress-induced suppression of LH, whereas the data we present in this study show that their activity is *sufficient* to cause a downregulation of pulsatile GnRH/LH. The role of endogenous RFRP-3 cannot be inferred from the present results obtained using stimulatory DREADDs. It should be highlighted that chemogenetic activation of RFRP neurons (or neurons that at 1 stage expressed *Rfrp*) led to a reduction in LH pulse frequency, not a complete cessation of it, and therefore they appear to lack the potency to completely clamp the GnRH pulse generator. These data corroborate our earlier findings, in which we demonstrated that chronically stimulating RFRP neurons does not cause any fertility impairments in male or female mice [3], suggesting RFRP-3 peptide is not necessary for reproductive function, but rather plays a permissive modulatory role.

The apparent sex-specific effects of *Rfrp*-Cre neuronal activation that we observed in this study is supported by others [3, 29], yet the mechanisms underpinning this sexual dimorphism remain unclear. Sex steroid milieu may play a permissive role, since RFRP neurons express estrogen receptor isoforms but not the androgen receptor [28]. We conducted all our experiments when female mice were in diestrus, so it might be interesting to see if the effects of endogenous RFRP neuronal activation on LH pulsatility are different during different cycle stages and/or hormonal milieus. While the precise mechanisms underlying the sex-specific role of RFRP neurons is unclear, its evolutionary purpose is easily envisaged. Reproduction is far more energetically costly for female mammals, so it makes sense that the regulation of female fertility has evolved to be more reactive and/or sensitive to any signal indicating that it is an inopportune time to reproduce, as we and others have recently reviewed [1, 2].

LH pulse dynamics in intact female mice show a lot of variability between individuals and throughout the estrous cycle [34], supporting a role for hormonal milieu contributing to the sexual dimorphism we observed. Interestingly, variability in LH pulse dynamics are also observed within a discrete cycle stage, as can be seen in our current and previous data [3], as well as in other experiments [34, 35]. Photometry data of synchronized episodes of kisspeptin neuronal firing, which is a hallmark of GnRH pulse generator activator and thus LH pulsatility, shows similar variability [36], so there are clearly other modulatory influences at play as well. Individual variability in response to handling and injections is a likely factor, despite the 2 weeks of habituation to this in the days leading up to sampling.

In conclusion, our data demonstrate that acute RFRP neuronal activation (whether in neurons that at one stage expressed *Rfrp* or in a subset of adult RFRP neurons) can independently cause a reduction in LH pulse frequency in female, but not male mice, without affecting LH pulse amplitude. Our results build on previous findings that RFRP-3 exerts a sex-specific “brake” on female, but not male, fertility [3, 29]. These data further demonstrate that RFRP neurons play a key role in the regulation of the reproductive axis.

## Data Availability

Some or all datasets generated during and/or analyzed during the current study are not publicly available but are available from the corresponding author on reasonable request.

## Abbreviations

DMH: dorsomedial nucleus of the hypothalamus
DREADD: designer receptor exclusively activated by designer drug
ELISA: enzyme-linked immunosorbent assay
GnRH: gonadotropin-releasing hormone
HA: hemagglutinin
HPG: hypothalamic–pituitary–gonadal
LH: luteinizing hormone
PBS: phosphate-buffered saline
RFRP-3: RFamide-related peptide-3
